# Original surgical treatment of thoracolumbar subarachnoid cysts in six chondrodystrophic dogs

**DOI:** 10.1186/1751-0147-56-32

**Published:** 2014-05-16

**Authors:** Camille Bismuth, François-Xavier Ferrand, Mathilde Millet, Philippe Buttin, Didier Fau, Thibaut Cachon, Eric Viguier, Catherine Escriou, Claude Carozzo

**Affiliations:** 1 Surgery Unit of the National Veterinary School of Lyon, VetAgroSup - Campus Vétérinaire de Lyon, Université de Lyon, 1 avenue Bourgelat, 69280 Marcy-l’étoile, France; 2 Neurology Unit of the National Veterinary School of Lyon, VetAgroSup - Campus Vétérinaire de Lyon, Université de Lyon, 1 avenue Bourgelat, 69280 Marcy-l’étoile, France

**Keywords:** Subarachnoid cyst, Leptomeningeal adhesion, Disc protrusion, Chondrodystrophic, Pug

## Abstract

**Background:**

Subarachnoid cysts are rare conditions in veterinary medicine, associated with spinal cord dysfunction. Most of the 100 cases of subarachnoid cysts described since the first report in 1968 were apparently not true cysts. Reported cysts are usually situated in the cervical area and occur in predisposed breeds such as the Rottweiler. The purpose of this retrospective study, from May 2003 to April 2012, was to describe the distinctive features of thoracolumbar spinal subarachnoid cysts, together with their surgical treatment and outcome in 6 chondrodystrophic dogs.

**Results:**

Five Pugs and 1 French Bulldog were examined. Images suggestive of a subarachnoid cyst were obtained by myelography (2/6) and computed tomography myelography (4/6), and associated disc herniation was observed in 3/6 dogs. A hemilaminectomy was performed. The protruding disc eventually found in 5/6 dogs was treated by lateral corpectomy. The ventral leptomeningeal adhesions observed in all dogs after durotomy were dissected. No or only mild post-operative neurological degradation was observed. Follow-up studies (7 months to 4 years) indicated good outcome and no recurrence.

**Conclusions:**

All the thoracolumbar subarachnoid cysts described in these 6 chondrodystrophic dogs were associated with leptomeningeal adhesions. Good results seemed to be obtained by dissecting and removing these adhesions. A protruding disc, found here in 5/6 dogs, needs to be ruled out and can be treated by lateral corpectomy.

## Background

Subarachnoid cysts (SAC) have been described as rare conditions in veterinary medicine, associated with spinal cord dysfunction, but seem to be increasing in frequency, with almost 100 new cases described since the first report by Gage et al. in 1968 [1]. A SAC is defined as a localised enlargement of the subarachnoid space with accumulation of cerebrospinal fluid (CSF). Numerous classifications and terminologies for SACs have been proposed in humans and animals. The following three categories have been proposed for human spinal SACs: type I: spinal extradural cysts without spinal nerve root fibre involvement; type II: spinal extradural cysts with nerve root involvement; and type III: spinal intradural meningeal cysts [2]. Canine SACs have been classified as type III. Communication with the adjacent subarachnoid space, determined after injecting contrast media into the subarachnoid space, may be rapid with free passage of contrast medium between the two (Category 1) or slow with delayed entrance and clearing of contrast medium (Category 2). Canine SACs fall into the first category [3,4]. However, none of the cysts reported in animals were lined with epithelial cells and therefore were not true cysts [3]. This suggests that the term “cyst” is actually incorrect and terms such as “leptomeningeal cavitation” or “dilatation” would be more appropriate [3,5]. In this report, the term “subarachnoid cyst” will be used to remain consistent with existing human and veterinary literature.

The distribution of canine spinal thoracolumbar SACs is mainly restricted to areas of high spinal mobility [3,6] and has been described in breeds such as the Rhodesian Ridgeback, Weimaraner, Shih Tzu, Shar Pei and Pug. Most SACs in humans are found on the posterior aspect of the spinal cord, but may, very rarely, develop in the cervical area [7], whereas in canine breeds they are often reported in the cervical area (C2-C5 (C: cervical)), particularly in the Rottweiler [3,5].

We carried out a retrospective study, between May 2003 and April 2012, in dogs that had been treated surgically for spinal thoracolumbar subarachnoid enlargement. Our main objectives were to describe the original features of the reported cases, e.g., leptomeningeal adhesions and disc protrusion, and the results of surgical treatments.

## Methods

Medical records of dogs, presenting between May 2003 and April 2012 at the National Veterinary School of Lyon with neurological signs of an upper motor neuron (UMN) lesion in the pelvic limbs, were reviewed and graded using Scott’s neurologic status scale [8]: grade 1: thoracolumbar pain with no neurological signs; grade 2: ataxia, conscious proprioceptive deficits and ambulatory paraparesis; grade 3: non-ambulatory paraparesis; grade 4: paraplegia with urinary retention and overflow (or with bladder control); and grade 5: paraplegia, urinary retention and overflow, and loss of deep pain sensation. To be included in the study, the SAC diagnosis had to have been confirmed by imaging and the SAC surgically addressed (Table 1).

**Table 1:** Dogs included in the study: description, findings and outcome

**Dog**	**Breed**	**Age (years)**	**Gender**	**Onset-duration**	**Clinical signs**	**Disc affected**	**Surgery**	**Hospitalisation**	**Outcome**
1	Pug	7	Male	2 months	Grade 3, worse on the left side.	T12-T13	Left hemilaminectomy T11-T13. Lateral corpectomy T12-T13.	3 days no degradation	Grade 2. Died 2 months later from unrelated cause (gastric perforation).
2	Pug	7	Female	3 months	Grade 2 then 3 with faecal incontinence.	T11-T12	Right hemilaminectomy T11-T12. Lateral corpectomy T11-T12.	3 days mild degradation	Grade 2. Lost to follow up 2 months after surgery.
3	French bulldog	0.5	Male	1 month	Grade 3, worse on the right side.	T11-T12	Left hemilaminectomy T9-T10-T11-T12. Lateral corpectomy T11-T12.	4 days mild degradation	Grade 2, worse on the right side 4 years after surgery.
4	Pug	9	Male	15 days	Grade 2, worse on the left side.	T11-T12	Left hemilaminectomy T10-T12. Lateral corpectomy T11-T12.	3 days no degradation	Grade 2, 8 months postoperatively.
5	Pug	6	Male	15 days	Grade 3.	T11-T12	Right hemilaminectomy T10-T12. Lateral corpectomy T11-T12.	3 days mild degradation	Grade 2 worse on the right side. Euthanasia for tetraplegia 2 years after surgery.
6	Pug	6	Female	3 months	Grade 3.	T7-T8	Left hemilaminectomy T7-T11.	2 days mild degradation	Grade 2.

T: Thoracic vertebra.

### Imaging diagnosis

Diagnosis was either based on survey radiographs and myelography or on CT myelography. Radiographs of the thoracolumbar spine were reviewed. Myelograms were obtained by subarachnoid injection of Iohexol (0.3-0.4 ml/kg of body weight) (Omnipaque®, Iohexol 300 mg/ml, Sanofi Winthrop Pharmaceutical, New York, NY) between the 5^th^ and 6^th^ lumbar vertebrae.

Cross-sectional computed tomography scans (CT scans) were obtained with a third generation scanner (GE High Speed NXI 2002, 2 slice CT Scanner, General Electric Healthcare, Chalfront St-Giles, RU). CT myelography (1 ml of Iohexol diluted in 1 ml of sterile saline) (Omnipaque®, Iohexol 240 mg/ml, Sanofi Winthrop Pharmaceutical, New York, NY) was performed by taking contiguous, 1-mm, transverse images of the entire spine. A three dimensional reconstruction was obtained from each CT scan using computer software (Osirix, 64bit, Pixmeo Swiss made, Geneva, Switzerland).

These imaging techniques were used to determine the position (relative to the spinal cord) and size of the lesions (length relative to the vertebrae) and to examine the vertebrae and discs adjacent to the lesions.

### Anaesthesia

For diagnostic imaging and for surgery, all dogs were anesthetised with either alfaxolone (Alfaxan® to effect, intravenously (IV)) or propofol (Propovet® to effect, IV), then intubated and maintained under general anaesthesia (Isoflurane) (ET 1–2.2%) in 100% oxygen and adjusted according to the anaesthetic depth. Lactated ringer solution (10 ml/kg/h IV) was administered throughout anaesthesia. Cephalexin (30 mg/kg IV) was administered prophylactically before surgery and again at the end of surgery. The dogs also received dexamethasone (0.1 mg/kg IV) before surgery. Pain was controlled with a morphine-lidocaine-ketamine drip (5 μg/kg/h-0.05 mg/kg/min-10 mg/kg/h, respectively, IV) during surgery and for the next 4 hours.

### Surgical technique

The thoracolumbar spine was approached dorsolaterally. The abnormal area was treated by hemilaminectomy [9] followed, in the case of disc protrusion, by lateral corpectomy [10]. The SAC was treated by dissecting the observed ventral leptomeningeal adhesions and by durectomy.

### Postoperative care

After surgery, the dogs’ neurologic status was monitored, and signs of pain were assessed by physical examination. Postoperative analgesia was ensured with morphine chlorhydrate (0.2 mg/kg subcutaneously (SC) or intramuscularly (IM) every 4 hours for 2 days) and buprenorphine (0.02 mg/kg SC every 8 hours for 2 days). Although the rules of antibiotic therapy in our institution have changed from that time i.e., only for contaminated neurosurgery (empyema etc.), cephalexin was continued for 10 days (15 mg/kg per os twice a day) in 3 dogs in which surgery time exceeded 2 hours and anaesthesia exceeded 3 hours (Dogs 1, 2 and 3). Corticosteroid therapy was continued by administering tapering doses of prednisolone i.e., 0.5 mg/kg PO twice a day for 5 days, then once a day for 5 days and finally every two days for 5 days. All dogs received standard physiotherapy during hospitalisation, which consisted of massage and passive movement of the limbs (stretching and bending) 3 times daily, starting on the day after surgery. Samples of the ventral adhesions were obtained from 3 dogs (Dogs 3, 4 and 6) and examined by the histopathology unit.

### Outcome

Follow-up data were obtained during the second examination 4 weeks after surgery. Owners and referring veterinarians were re-contacted by telephone (i.e., from 7 months to 4 years after surgery) to assess the current disease status, particularly whether the dog was walking or not, and the owner’s satisfaction regarding the improvement of neurological status.

Due to the small number of dogs in the study, the results could not be subjected to statistical analysis or compared with other treatments.

## Results

### Case descriptions

Six dogs were included in the study. All were chondrodystrophic dogs – 5 Pugs and 1 French Bulldog. Included were 4 males and 2 females, with a mean age of 5.9 years (0.5 to 9 years). Neurological signs were always related to UMN lesions, with pelvic limb ataxia (2/6 dogs) and paraparesis (4/6 dogs), associated in 1 case with faecal incontinence (Dog 2); these signs were of 15 days to 3 months duration and showed slow progression.

**Figure 1:**
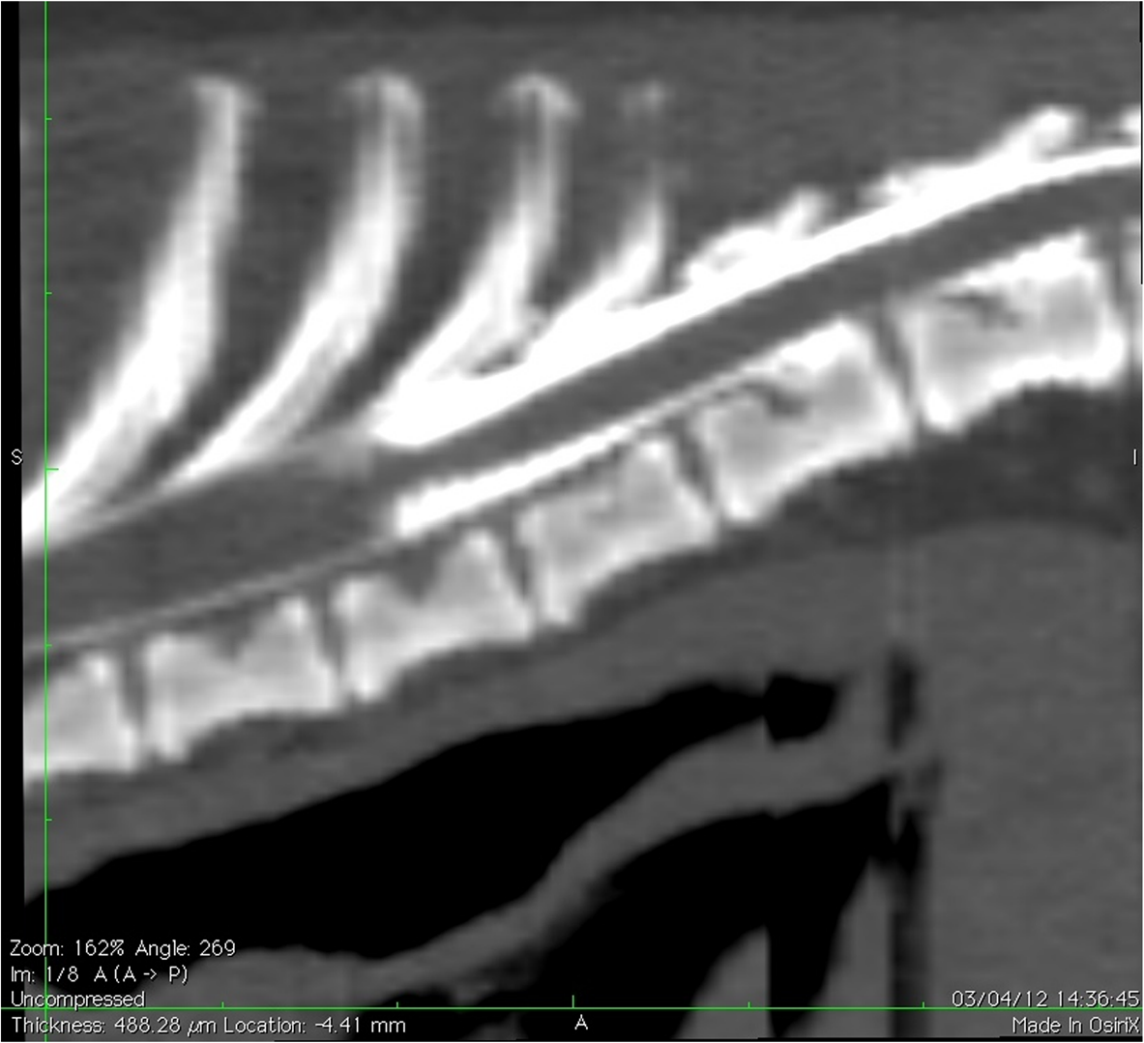
**Sagittal plane CT myelogram.** CT image showing the focal accumulation of contrast medium with a “tear-drop” shape (sagittal plane) (Dog 3).

**Figure 2:**
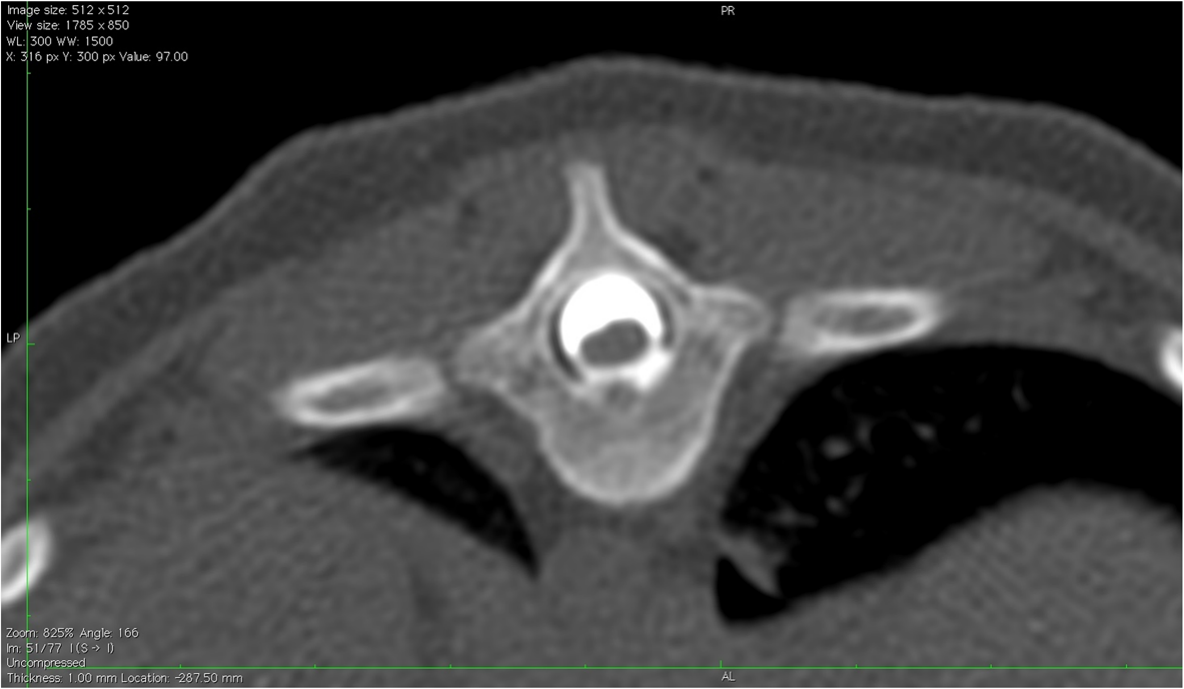
**Transverse plane CT myelogram.** CT image showing the focal accumulation of contrast medium in transverse plane. The spinal cord is ventrally deviated (Dog 6).

Myelography was carried out in 2/6 dogs (Dogs 1 and 2) and CT myelography in 4/6 dogs (Dogs 3, 4, 5 and 6). The images of all dogs revealed a focal accumulation of contrast medium in the subarachnoid space, starting gradually and ending abruptly with a “tear-drop” enlargement on the lateral or sagittal views (Figures 1 and 2). The spinal cord was ventrally deviated and appeared thinner next to the lesion in all dogs. The CT images confirmed a slight reduction of spinal cord diameter but no evidence of an associated intramedullary lesion. No parenchyma enhancement was observed after the injection of contrast medium. The SAC was situated dorsally to the spinal cord extending from the 11^th^ to 12^th^ thoracic vertebrae in Dog 2, from the 10^th^ to 12^th^ thoracic vertebrae in Dog 4 and from the 13^th^ thoracic vertebra to the 2^nd^ lumbar vertebra in Dog 5. It was situated dorsolaterally on the left side of the 12^th^ vertebra in Dog 1, extended from the 9^th^ to 12^th^ thoracic vertebrae in Dog 3 and from the 7^th^ to 8^th^ thoracic vertebrae in Dog 6.

Diagnostic imaging revealed a disc protrusion in 3/6 dogs (Dogs 1, 4 and 5). In Dog 1, the disc between the 11^th^ and 12^th^ thoracic vertebrae was more opaque, protruded slightly into the spinal canal on the left side and was associated with some spondylosis of the caudal plate of the 12^th^ vertebra. In Dogs 4 and 5, the ventral column was dorsally deviated in relation to the intervertebral space, compatible with a disc protrusion, between the 11^th^ and 12^th^ thoracic vertebrae and between the 13^th^ thoracic vertebra and the 1^st^ lumbar vertebra, respectively. Some vertebral spondylosis was observed between these latter vertebrae in Dog 4. No obvious presence of a herniated disc was detected in Dogs 2, 3 and 6.

No abnormality of the vertebral column was found in any dog in the study.

### Surgical technique

**Figure 3:**
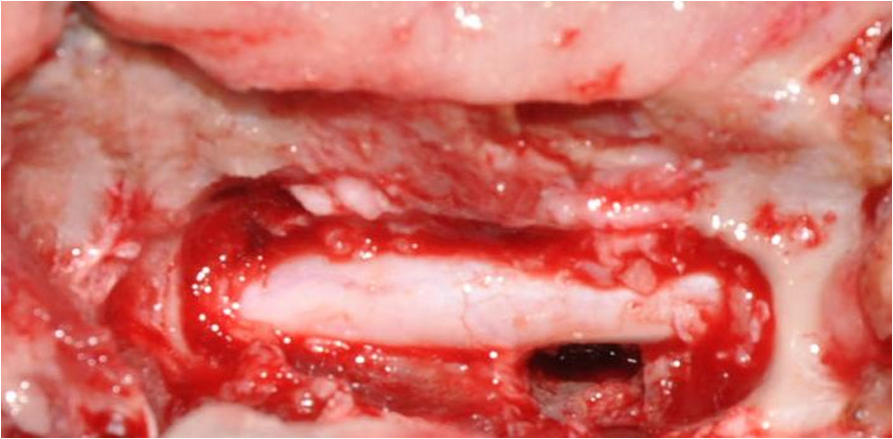
Lateral corpectomy (Dog 4).

**Figure 4:**
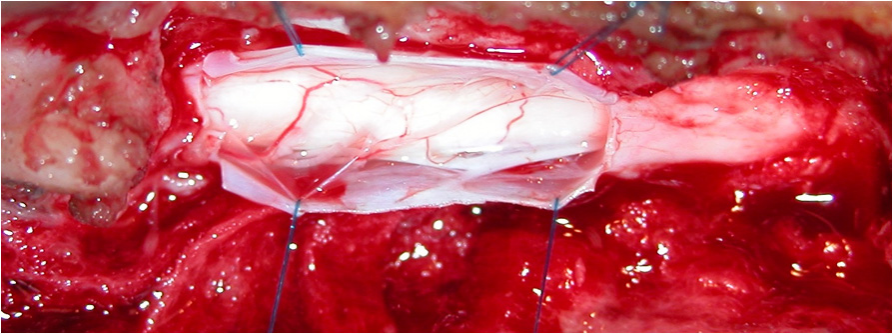
**Leptomeningeal adhesions.** Visualisation of the ventral leptomeningeal adhesions between the pia mater and the arachnoid to pia mater (Dog 5).

**Figure 5:**
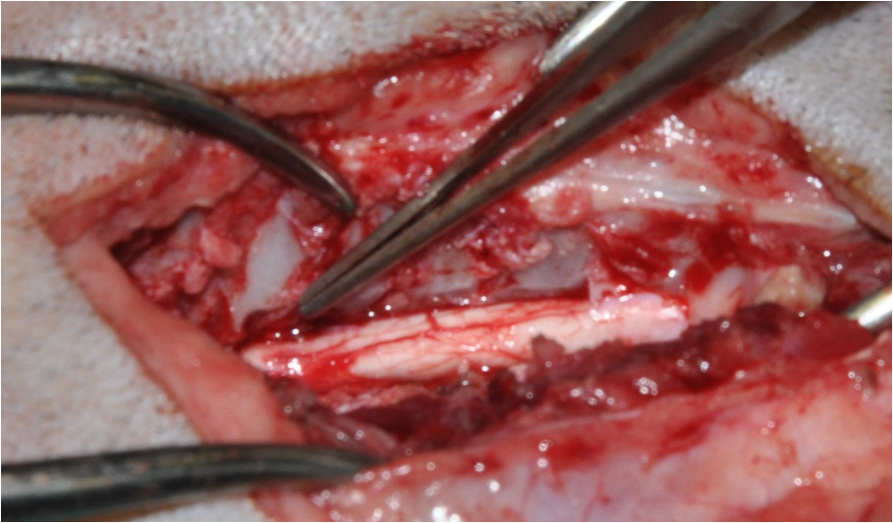
**Persistence of a dorsal mark.** Persistence of a slight dorsal mark (tip of the forceps) at the level of the subarachnoid cyst after dissection of the ventral leptomeningeal adhesions (Dog 6).

**Figure 6:**
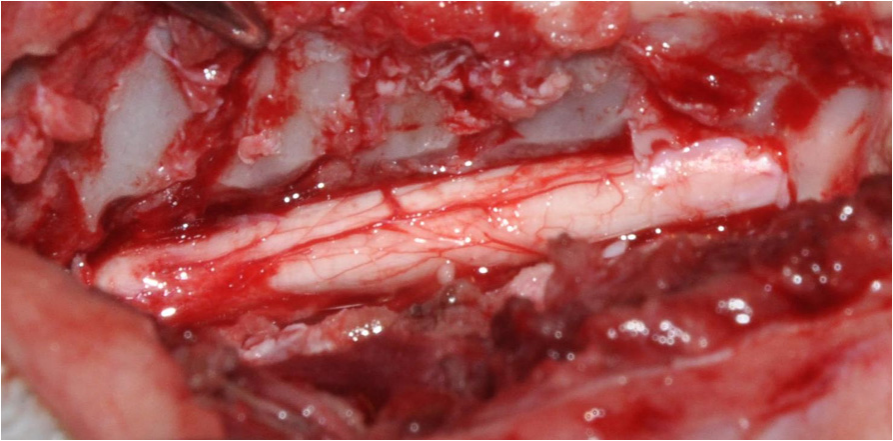
**Durectomy.** Durectomy (3/4 of the diameter of the dura mater) (Dog 6).

Surgery consisted of 3 different steps, depending on the observed lesions. First, a hemilaminectomy was performed in all dogs. The disc was then assessed with a neurosurgical ballpoint probe. Disc protrusion, when present, was treated by lateral corpectomy (Figure 3) [10]. The presence of a protruding disc was not obvious in Dogs 2 and 3 during imaging diagnosis but became evident during surgery when an abnormal bulge of the annulus fibrosus was readily palpable with a neurosurgical ballpoint probe. The protrusion was evident during imaging diagnosis and surgery in 3 other dogs (Dogs 1, 4 and 5). Dog 6 did not show any protruding disc during either CT myelography or surgery. However, a small ‘step’ in relation to the SAC, between the 7^th^ and 8^th^ thoracic vertebrae, could be felt with a neurosurgical ballpoint probe. This abnormality did not require surgical correction. Finally, a durotomy with dural suspension was performed in all dogs on the side of the hemilaminectomy. In all cases, the dura mater was thickened and a large volume of colourless CSF was obtained after fenestration. A depression of the spinal cord was apparent. Ventral adhesions between the dura mater and the pia mater and arachnoid were observed in all cases (Figure 4) but were not visible dorsally. The dura mater and the arachnoid from the pia mater were dissected under optical magnification (surgical microscope or magnifying glasses) over half to two thirds of the ventral circumference of the spinal cord until no impediment could be felt with a neurosurgical ballpoint probe over the entire area. This part of the dura mater was removed by durectomy. The ventral deviation of the spinal cord appeared to be consistently attenuated in all dogs after dissection of the adhesions, whereas it persisted after opening of the SAC. However, in all cases, a slight dorsal mark persisted on the spinal cord at the level of the subarachnoid cyst (Figure 5). The dura mater above the cyst was removed on the side of the hemilaminectomy. The estimated extent of the durectomy (at the level of the adhesions and cyst) ranged from almost 2/5 to 1/2 of the total dura mater circumference (Figure 6). Marsupialisation was not performed. The surgical field was closed routinely with apposition of either a free fat graft (Dogs 2, 3, 4 and 6), or a non-adherent biofilm (Dogs 1 and 5) (Seprafilm®, Genzyme SAS, Sanofi, USA) to the exposed spinal cord.

The mean anaesthesia time was 3 hours (2.5-3.5 hours) and, on average, surgery was completed within 2 hours (1.5-2.5 hours).

Histopathologic analyses of the tissue removed during surgery from Dogs 1, 4 and 6 revealed connective tissue proliferation with fibrosis.

### Outcome

The mean duration of hospitalisation was 3 days (ranging from 2 to 4 days) with no or only mild degradation of neurologic status (ataxia to paraparesis).

Postoperative follow-up, 7 months to 4 years after surgery, revealed that the owners of 5/6 dogs were satisfied with their dog’s status of ambulation with intermittent hind limbs weakness (Table 1). Dog 1 showed neurological improvement but died from a gastric perforation, which was a suspected complication of steroid treatment 2 months after surgery. Dog 5 was euthanised 2 years after surgery for onset of acute tetraplegia. The postoperative outcome, in relation to this study, was considered good by the owner of Dog 5, as the dog was able to walk before the acute tetraplegia.

## Discussion

Leptomeningeal adhesions, resulting from chronic microtraumas associated with vertebral instability or with features associated with vertebral instability (such as a protruded disc), can further enlarge the subarachnoid space and could have been a significant factor in spinal cord compression and dysfunction in our 6 chondrodystrophic dogs. Such SACs should therefore be treated by removing the leptomeningeal adhesions and the source of adhesive arachnoiditis, and not just by opening and draining the SAC.

Approximately 100 cases of canine SACs have been described in the veterinary literature. Cervical SACs are particularly common in Rottweilers, which are more represented than other breeds [3,5,10]; lumbar SACs are common in Rhodesian Ridgebacks due to the importance of spinal dysraphism and neural crest fusion failure in this breed [11-13]; and thoracolumbar SACs are seen in Pugs [11-13]. All the dogs in our study were chondrodystrophic, i.e., 5 Pugs and 1 French bulldog, and had thoracolumbar lesions. Only 15 of the 147 Pugs seen at our practice during the study period were brought in for neurological signs. Two exhibited a vertebral column malformation, 5 had a chronic thoracolumbar disc protrusion not associated with a SAC, 3 had an acute thoracolumbar disc extrusion and 1 had an acute cervical disc extrusion. This may indicate a possible predisposition of Pugs to thoracolumbar SACs (5/15 dogs in 9 years).

All the dogs in our group were examined for vertebral malformations, as these abnormalities have been described in Pugs [14] and French bulldogs. However, radiographic examinations and CT scans, which included 3D reconstructions of CT scans with particular emphasis on articular facets (i.e., aplastic or hypoplastic articular facets [14]), did not reveal any vertebral abnormalities in the SAC area. Nevertheless, the possibility of vertebral column instability was retained, as disc protrusion was observed in 5/6 dogs, with signs of spondylosis in 2/5 dogs, and a small subluxation was detected in Dog 6. Although no gender predisposition has been reported, some studies mentioned that males were over-represented, as in our study (ratio 4:2) [3]. The predisposition of males has been established in humans, but remains unexplained [3].

Faecal or urinary incontinence (1/6 dogs) are common clinical findings in thoracolumbar SACs, associated with upper motor neuron pelvic limb ataxia (2/6 dogs) and paresis (4/6 dogs) [6]. These clinical signs seem to be related to the frequent dorsal localisation of SACs and interruption of the sensitive urinary and faecal continence pathway [15].

Imaging diagnoses revealed that the SACs in our study were typical i.e., single and dorsal or dorsolateral [3,5,6,11,12,16]. Both imaging techniques utilised in this study are commonly used in veterinary medicine and can provide a reliable confirmation of a SAC diagnosis [5,6,13]. CT myelography, compared to myelography, can provide additional information about the extent of the lesion as well as spinal cord abnormalities such as syringohydromyelia or flattening, irregularity, atrophy or misshapenness of the spinal cord [5,13,20]. Survey radiographs can be used to detect SACs in humans, but not in dogs [12,13,15-17]. Finally, magnetic resonance imaging (MRI) provides better contrast resolution and anatomical definition and is therefore a preferred choice for diagnosing subarachnoid enlargement and fine characterisation of the cyst contents [3,20]. MRI was not used in our study mainly because our primary hypothesis, for all but one dog (Dog 3), was a herniated disc. Due to the young age of this dog, a skeletal abnormality was also likely.

The aetiology of canine SACs is still unclear. It was initially suggested that they might be induced by prior chronic inflammatory reactions of the arachnoid, known as arachnoiditis, as in humans [1]. However, veterinary reports, based on examination of the cerebrospinal fluid (i.e., normal) or histology of the dura mater and arachnoid above the SAC (i.e., fibrous tissue or normal meninges), have not confirmed this [3,5,6,13,16,18-21]. The results of our histopathologic analysis were consistent with the observations above.

The possibility of acute trauma or repetitive trauma to the spinal cord was then examined, as for cases of vertebral instability or disc protrusion in humans [1,3,6,13,20]. Such trauma may lead to adhesive arachnoiditis and ventral leptomeningeal adhesions, as observed in our dogs, which can then create an enlargement of the subarachnoid space and an accumulation of cerebrospinal fluid on the side opposite to the leptomeningeal adhesions. The passive accumulation of CSF without pressure, as in a cistern or a lake, has already been described in dogs [3].

Therefore, one possible aetiology proposed for high cervical and thoracolumbar SAC formation in dogs is vertebral column instability, leading to chronic meningeal microtraumas and adhesive arachnoiditis [1,3,6]. This is in agreement with the histopathologic observations of the ventral dura mater and arachnoid (i.e., ventral adhesions) in 3/6 dogs in our study, and in 10 dogs in another study [3], which indicated connective tissue proliferation and fibrosis with no or moderate inflammation. The role played by chronic meningeal microtraumas, due to vertebral column instability or features associated with such instability, has been demonstrated in C2-C3 instability [3,22], caudal cervical spondylomyelopathy [6] and vertebral deformity [15,23]. Disc protrusion had already been observed in association with SACs, but not in the same space or only after the SAC had been opened during hemilaminectomy or laminectomy in 4 dogs [5,6,15]. The chronic pathology of the SACs in our study coincides with an average age of 5.9 years, even if numerous reports of SACs have involved young dogs, when considering all vertebral sites taken together, and therefore suggests a possible inherited aetiology or congenital pathology [3,6,11,24]. It seems that dogs with cervical cysts tend to be young, large-breed dogs, such as Rottweilers, with SACs and suspected congenital C2-C3 instability; whereas dogs with spinal caudal thoracic cysts are typically small breeds and are diagnosed later in life, like the dogs in our study [3,6,11]. Moreover, the potential role of chronic meningeal microtraumas is supported by the fact that, according to the owners, the neurologic signs appeared 15 days to 3 months before surgery and progressed slowly [5,6,11,16].

Imaging diagnoses revealed a disc protrusion in only 3/6 dogs. Myelography and CT scanning are known to facilitate the accurate diagnosis of a herniated disc, even if CT scanning is considered more sensitive than myelography for detecting lesions in chronically affected dogs [25]. CT myelography seems to be even more accurate than CT scanning [26] and is routinely employed in our facilities in the case of suspected herniated disc. In this study, the presence of a herniated disc was diagnosed in 2/3 cases by CT myelography and in only one out of two dogs by myelography. To our knowledge, the likelihood of accurately detecting both lesions at the same time, as in our dogs, has not been assessed.

The possible presence of chronic disc protrusion is worth investigating during surgery. In fact, due to the surgeon’s experience, a disc protrusion was actually detected during surgery in 5/6 of our dogs, which underlines the potential role played by disc herniation in SAC pathology. Dog 6, however, did not show any sign of disc protrusion, and the SAC was localised between the 7^th^ and 8^th^ thoracic vertebrae, a region not predisposed to herniated discs. Instead, a ‘step’ could be felt between the 2 vertebrae, which suggests that the ventral leptomeningeal adhesions in this dog might have been due to transient vertebral column instability that resulted in a small static subluxation, without any evidence of spondylosis. Vertebral distraction and stabilisation were considered unnecessary because of the small size of the step and the apparent perioperative stability of the two vertebrae.

We cannot assert with certainty that chronic disc protrusion was actually responsible for the observed thoracolumbar leptomeningeal adhesions as both lesions can occur independently, particularly in areas of increased mobility (85% between T11-T12 and L2-L3 (T: thoracic; L: lumbar)) and in middle-aged dogs (5–8 years) [3,8,18,24]. It is interesting to note that chondrodystrophic dogs are generally predisposed to disc extrusion rather than to disc protrusion. In our experience, Pugs can exhibit both, as 5 of the 15 pugs examined over 9 years were for disc protrusion and 4 were for disc extrusion. As both lesions are primary causes of spinal cord compression, it seems appropriate to address the SAC “aetiologically” and to consider both possibilities during surgery. Because of the highly probable link between chronic meningeal microtraumas, adhesive arachnoiditis and SAC, we chose not only to open the SAC, as previously described, but also to remove the leptomeningeal adhesions, as in the study by Gnirs et al. [3,27]. A ventral deviation of the spinal cord has consistently been reported, in fact, even after marsupialisation or durectomy [16,21]. This ventral deviation was noted before all of our surgical interventions but was apparently reduced after dissection and removal of the ventral leptomeningeal adhesions, with no macroscopic evidence of spinal cord oedema. In each case, only a slight dorsal mark remained at the level of the subarachnoid cyst (Figure 5).

Reported outcomes following the current surgical treatment of SACs (i.e., partial durectomy or marsupialisation via hemilaminectomy or dorsal laminectomy), have been good (both in the short- and long-term) when associated disc protrusion is absent. Some authors, however, believe that long-term outcomes are trending towards a worsening of clinical signs [3,5,6,13,28]. Worsening of the neurologic status and the recurrence of neurologic signs are still current concerns, and a 20% rate of recurrence was reported in the study by Skeen et al. [6]. In the present study, all dogs showed neurological improvement 4 weeks after surgical treatment, which included dissection of the leptomeningeal adhesions and a lateral corpectomy, and no neurological recurrence was reported by the owners. However, due to the retrospective nature of the study and the small number of cases, it was not possible to include a control group (i.e., marsupialisation vs durectomy alone, lateral corpectomy vs no lateral corpectomy) or to objectively assess the neurologic status before and after surgery (i.e., with force plates). More studies are therefore needed to confirm the contribution of completely dissecting the leptomeningeal adhesions and performing a lateral corpectomy in such dogs.

The protruding disc was treated by lateral corpectomy because, in dogs with disc protrusion and therefore chronic disc herniation, the protruded material is fibrous or fibrocartilaginous in structure and firmly attached to the remaining annulus fibrosus [10]. It could be argued that combining a hemilaminectomy with lateral corpectomy, as in this study, might create a potential source of vertebral column instability, as demonstrated by Vizcaíno Revés et al. [29]. However, because this latter study was performed in vitro, the musculature of the vertebral column was not taken into account and there was no comparison with hemilaminectomy alone. No instability was detected in the previous clinical and biomechanical studies [10,30,31], and we are not currently aware of any reports of this complication occurring with subsequent clinical consequences in dogs.

The limitations of this study include its retrospective nature and the small number of cases. More retrospective and prospective studies are therefore needed to confirm, or not, the predisposition of chondrodystrophic dogs (particularly Pugs) to the development of SACs associated with a disc protrusion, the role of leptomeningeal adhesions in SAC development, the role of chronic herniated discs in the creation of leptomeningeal adhesions, the interest of addressing a SAC alone or in association with leptomeningeal adhesions and, finally, the benefit of including a lateral corpectomy when a concomitant herniated disc is present.

## Conclusions

The thoracolumbar subarachnoid cysts described in 6 chondrodystrophic dogs were all associated with leptomeningeal adhesions, meaning that the subarachnoid cyst might not necessarily be the direct cause of spinal cord compression in these dogs. An associated disc protrusion has to be considered, particularly in chondrodystrophic dogs, as this was observed in 5 out of 6 dogs in our study. Even though the dogs in the present study were able to walk again and the owners considered the outcome to be satisfactory, more studies are needed to confirm the benefit of removing the leptomeningeal adhesions and including lateral corpectomy in the case of disc protrusion.

## Abbreviations

SAC: Subarachnoid cyst; CSF: Cerebrospinal fluid; C: Cervical; UMN: Upper motor neuron; CT: Computed tomography; IV: Intravenously; SC: Subcutaneously; IM: Intramuscularly; MRI: Magnetic resonance imaging; T: Thoracic; L: Lumbar.

## Competing interests

The authors declare that they have no competing interests.

## Authors’ contributions

DF, TC, and EV participated in the case recruitments, surgeries, hospitalisations and follow-ups. FXF, MM and PB helped with surgery and took part in hospitalisations and follow-ups. CE participated in the case recruitments and early drafting of the manuscript. CB took part in surgery, hospitalisations, follow-ups, data collection and wrote the final draft of the manuscript. CC generated the objectives, participated in case selection, surgery, follow-ups and corrected the final draft of the manuscript. All authors read and approved the final version of the manuscript.
